# Protective Effect of Chitin Urocanate Nanofibers against Ultraviolet Radiation

**DOI:** 10.3390/md13127076

**Published:** 2015-12-19

**Authors:** Ikuko Ito, Toshikazu Yoneda, Yoshihiko Omura, Tomohiro Osaki, Shinsuke Ifuku, Hiroyuki Saimoto, Kazuo Azuma, Tomohiro Imagawa, Takeshi Tsuka, Yusuke Murahata, Norihiko Ito, Yoshiharu Okamoto, Saburo Minami

**Affiliations:** 1 Department of Veterinary Clinical Medicine, Tottori University, Tottori 680-8553, Japan; i.ikuko95@gmail.com (I.I.); kazu-azuma@muses.tottori-u.ac.jp (K.A.); imagawat@muses.tottori-u.ac.jp (T.I.); tsuka@muses.tottori-u.ac.jp (T.T.); ymurahata@muses.tottori-u.ac.jp (Y.M.); taromobile@me.com (N.I.); yokamoto@muses.tottori-u.ac.jp (Y.O.); saburominami@ncn-t.net (S.M.); 2 Omura Toryo Co., Ltd., 87, 3-Chome, Chiyomi, Tottori 680-0991, Japan; omurapar@apionet.or.jp (T.Y.); omurap@apionet.or.jp (Y.O.); 3 Graduate School of Engineering, Tottori University, Tottori 680-8552, Japan; sifuku@chem.tottori-u.ac.jp (S.I.); saimoto@chem.tottori-u.ac.jp (H.S.)

**Keywords:** chitin nanofibrils, urocanic acid, UVB, erythema, sunburn cell

## Abstract

Urocanic acid is a major ultraviolet (UV)-absorbing chromophore. Chitins are highly crystalline structures that are found predominantly in crustacean shells. Alpha-chitin consists of microfibers that contain nanofibrils embedded in a protein matrix. Acid hydrolysis is a common method used to prepare chitin nanofibrils (NFs). We typically obtain NFs by hydrolyzing chitin with acetic acid. However, in the present study, we used urocanic acid to prepare urocanic acid chitin NFs (UNFs) and examined its protective effect against UVB radiation. Hos: HR-1 mice coated with UNFs were UVB irradiated (302 nm, 150 mJ/cm^2^), and these mice showed markedly lower UVB radiation-induced cutaneous erythema than the control. Additionally, sunburn cells were rarely detected in the epidermis of UNFs-coated mice after UVB irradiation. Although the difference was not as significant as UNFs, the number of sunburn cells in mice treated with acetic acid chitin nanofibrils (ANFs) tended to be lower than in control mice. These results demonstrate that ANFs have a protective effect against UVB and suggest that the anti-inflammatory and antioxidant effects of NFs influence the protective effect of ANFs against UVB radiation. The combination of NFs with other substances that possess UV-protective effects, such as urocanic acid, may provide an enhanced protective effect against UVB radiation.

## 1. Introduction

### 1.1. DNA Damage and Skin Damage by UV Light

Ultraviolet (UV) radiation has a harmful effect on the human body. UV radiation can be classified according to wavelength as UVA (320–380 nm), UVB (290–320 nm), and UVC (210–290 nm). Because the ozone layer blocks UVC radiation, only UVA and UVB reach the earth’s surface. UV radiation with shorter wavelengths has higher energy and is more likely to cause skin damage. Exposure to UVB causes DNA damage and dermatopathies such as inflammation, pigmentation, skin aging, photoallergy, skin cancer, and immune dysfunction [1,2]. The mechanism underlying DNA damage has been determined as follows: DNA has a maximum absorption at approximately 260 nm; therefore, UVC and some UVB radiation of certain wavelengths can cause DNA nucleotides to absorb energy, which results in their transition to an excited state. Molecules in such an excited state are unstable, and in order for them to return to a stable state, new chemical bonds are formed. These differ from the original bonds, and this shift damages the DNA [1]. In addition, DNA damage also occurs as a result of irradiation with UVA or UVB rays within a range of wavelengths where there is no UV absorption. This is thought to be due to reactive oxygen species (ROS), which are generated as a result of irradiation [3]. Chromophores such as NADH and FAD, which absorb UV rays, are present in the cell and are activated by light energy. As a result, ROS, such as O_2_^−^, H_2_O_2_, and ^1^O_2_, are generated from oxygen. These ROS cause lipid peroxidation, protein polymerization or cleavage, enzyme deactivation, and various types of DNA damage. The potential DNA damage includes the production of thymine glycol due to thymine peroxidation as well as the production of uracil due to cytosine deamination [1]. In addition, ROS attack adenine and guanine, which causes cleavage of the imidazole ring and oxidation of guanine. DNA strands can also break. Such DNA damage is difficult to repair [1].

UVB-induced cutaneous damage leads to erythema, one of the cardinal signs of inflammation. Non-steroidal anti-inflammatory drugs (NSAIDs) suppress the early stages of erythema very well; however, their suppressive efficacy gradually weakens after the initial stage, regardless of whether or not the skin is treated with additional NSAIDs. It has been suggested that prostaglandins (PGs) might be responsible for the early phase of erythema that is depressed by NSAIDs; however, other factors may be involved in prolonging the duration of erythema [4]. Sunburn begins several hours after exposure, and flaring of the skin, swelling, and bullous formation peak 12–24 h later [1]. In the present study, the skin was observed for up to 24 h after exposure, when erythema peaks.

One in five Americans suffers from skin cancer [5], for which UV irradiation is the primary cause [6]. Risk of skin cancer is influenced by UV irradiation and skin pigmentation [7]. Hence, UV ray-blocking experiments using UVB irradiation have been performed by many scientists. Yasuoka *et al.* irradiated the back skin of hairless mice with 5 kJ/m^2^ UVB irradiation [8], and Mizukoshi *et al.* used 100 mJ/cm^2^ UVB irradiation to evaluate irradiation blocking ability [9]. By those various experiments, various sun protection materials are widely manufactured and used. The proof of these materials’ efficacy is of great significance for the protection of public health, as the UVB rays of solar radiation are the main contributors to sunburn, immunosuppression, and skin cancer [10]. According to Ananthaswamy *et al.*, application of SPF-15 sunscreen to mouse skin before each UV irradiation nearly eliminated p53 mutations, which play an essential role in skin cancer development [11].

In recent years, the demand for new, more functional sun protection materials have increased as the risk of the UV exposure has risen due to depletion of the ozone layer.

### 1.2. About UCA and Squid Ink as Positive Controls

The breakdown of filaggrin into hygroscopic free amino acids and their derivatives is the major contributor to the natural moisturizing factor (NMF) that is produced within the stratum corneum [12]. NMF is responsible for maintaining skin hydration and water retention within the stratum corneum under conditions of low environmental humidity [12]. Filaggrin is particularly rich in histidine. Histidine is deiminated into *trans*-urocanic acid (*trans-*UCA) by the catalytic action of histidase, and presumably provides a major source of epidermal UCA [13,14].

UCA is an intermediate metabolite of histidine and is present mainly in the stratum corneum. Urocanic acid is a major UV-absorbing chromophore in the skin [15]. *trans-*UCA is a major UVR-absorbing skin molecule that undergoes a photoisomerization to its *cis*-isomer following UVR exposure. *cis*-UCA has been shown to have immunosuppressive properties, whereas *trans*-UCA may act as a natural sunscreen due to its UV-absorbing properties [16]. *trans-*UCA has also been reported to inhibit UVB-induced secretion of IL-6 and IL-8 in the cornea [17], and it is an important protective factor against UV radiation.

Squid ink is comprised of eumelanin, a well-known natural UV ray absorber. The beneficial effects of melanin are mainly due to the presence of eumelanin, which scatters and absorbs 50%–75% of UVR. Eumelanin also scavenges UV-generated free radicals, which protect against UVR damage in deeper layers [18]. Therefore, we decided to use squid ink as a positive control.

### 1.3. About Chitin and Chiti Nanofibers

Chitins are highly crystalline structures that are found predominantly in crustacean exoskeletons, insects, cuttlefishes, shellfishes, mushrooms, and fungi. Alpha-chitin is composed of microfibers that are comprised of nanofibrils (NFs), which are approximately 2–5 nm in diameter and 30 nm in length and embedded in a protein matrix [19,20]. Isolated chitin NFs have potential for use in drug delivery systems, tissue-engineering scaffolds, and wound dressings [21]. We have recently succeeded in isolating alpha-chitin nanofibers from crab shells by a simple process [22]. An acidic condition is key to fibrillating the chitin effectively. A small number of amino groups in the chitin are cationized by the addition of an acid, which promotes the fibrillation of chitin into nanofibers by electrostatic repulsion.

NFs are thought to have great potential for various applications because they have several useful properties, including high specific surface area and high porosity [23,24]. Many studies have demonstrated the potential applications of chitin and chitosan NFs in tissue engineering, as wound dressings, in cosmetic and skin products, in stem cell technology, as anti-cancer treatments, as drug delivery systems, and as treatments for obesity and inflammatory conditions [25]. Until now, we have obtained NFs by hydrolyzing chitin with acetic acid.

In this study, we prepared UCA chitin NFs (UNFs) by UCA hydrolysis. We hypothesized that we could generate valuable new NFs with UV-blocking function by preparing NFs with UCA, and we examined the protective effect of the UNFs against UV radiation.

## 2. Results and Discussion

### 2.1. Erythema Score

All of the groups were irradiated (150 mJ/cm^2^) with UV (302 nm). In accordance with the criteria established by the OECD, erythema of the skin was assessed at 2, 4, 6, 12, and 24 h after irradiation.

**Figure 1:**
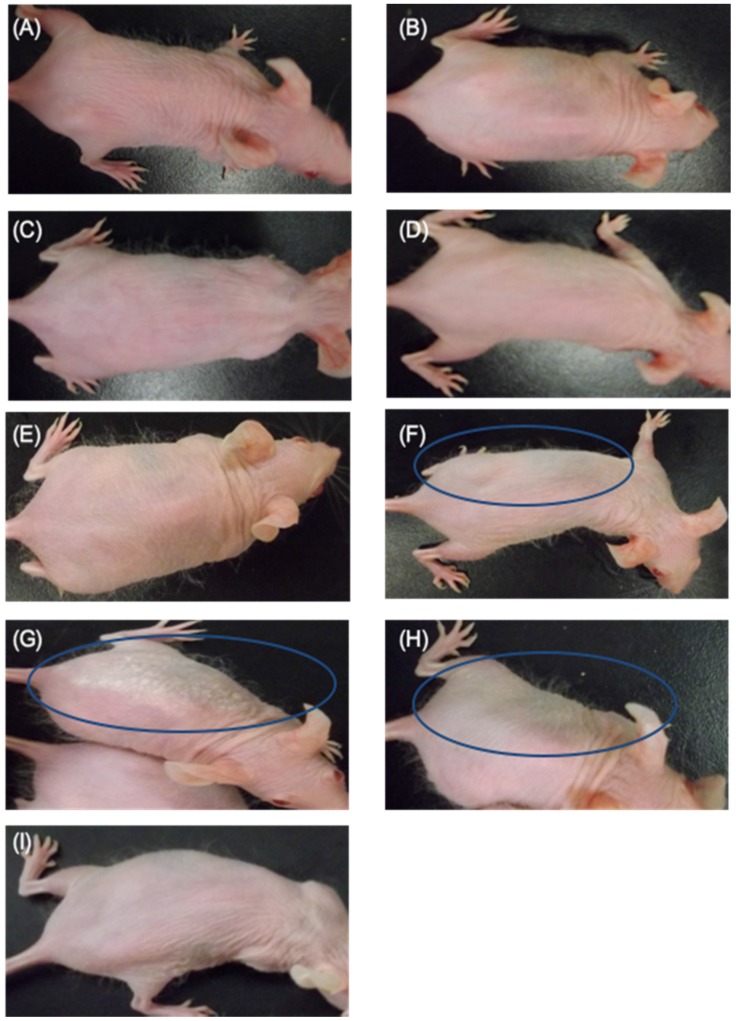
Erythema in hairless mice 12 h after UV irradiation (302 nm, 150 mJ/cm^2^). (**A**) C (+) (non-coated, irradiated) group; (**B**) HO (hydrophilic ointment)-coated group; (**C**) PG (polyethylene glycol) cream-coated group; (**D**) Ac (aqueous acetic acid) cream-coated group; (**E**) ANFs (acetic acid chitin nanofibrils) cream-coated group; (**F**) UCA (aqueous urocanic acid) cream-coated group; (**G**) UNFs (urocanic acid chitin nanofibrils) cream-coated group; (**H**) SI (squid ink) cream-coated group; and (**I**) C (−) (non-coated and non-irradiated) group. For **B**–**H**, each sample was applied to the left side of the mouse’s back. The color of the skin on the right and left sides of the mice in the UNA cream group, UCA cream group and Si cream group differed. Contrary to the findings in the C (+) group, UV-irradiation-induced erythema was markedly inhibited in the UNFs cream group as compared to the Si cream group, which was the positive control. The erythema of the UCA cream group was mildly inhibited. The erythema scores for the HO cream, PG cream, Ac cream, and ANFs cream groups were equivalent to the C (+) group. The places surrounded with an oval are the places where erythema is characteristically inhibited.

As shown in Figure 1, compared to the C (+) group, UV radiation-induced erythema was markedly reduced in the SI cream group. The erythema score for the C (+) group was similar to the erythema scores for the HO, PG cream, Ac cream, and ANFs cream groups. In addition, compared with the C (+) group, UV irradiation-induced erythema was markedly inhibited in the UNFs cream group as compared to the Si cream group, which was the positive control. Erythema in the UCA cream group was mildly inhibited.

**Figure 2:**
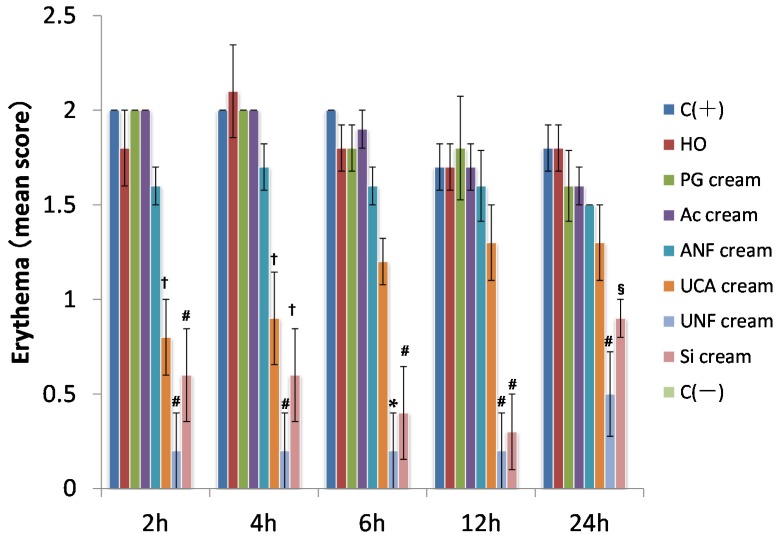
Time-course measurement of cutaneous erythema induced by UV irradiation (302 nm, 150 mJ/cm^2^). C (+) (non-coated, irradiated) group; HO (hydrophilic ointment)-coated group; PG (polyethylene glycol) cream-coated group; Ac (aqueous acetic acid) cream-coated group; ANFs (acetic acid chitin nanofibrils) cream-coated group; UCA (aqueous urocanic acid) cream-coated group; UNFs (urocanic acid chitin nanofibrils) cream-coated group; SI (squid ink) cream-coated group; and C (−) (non-coated and non-irradiated) group. * *p* < 0.01, significantly different from C (+), HO, PG, Ac, ANFs, and UCA. **^#^** *p* < 0.01, significantly different from C (+), HO, PG, Ac, and ANFs. **^†^** *p* < 0.01, significantly different from C (+), HO, PG, and Ac. ^§^ *p* < 0.01, significantly different from C (+) and HO. The error bars indicate mean ± SE. (Scheffé’s *F*-test).

In Figure 2, the degree of erythema in each group is shown as a score. Two hours after UV irradiation, the erythema scores for the UNFs cream and SI cream groups were significantly lower than those of the C (+), HO, PG cream, Ac cream, and ANFs cream groups. The erythema scores for the UCA cream group were significantly lower than those of the C (+), HO, PG cream, and Ac cream groups.

Four hours after UV irradiation, the erythema score for the UNFs cream group was significantly lower than the scores for the C (+), HO, PG cream, Ac cream, and ANFs cream groups. The erythema scores for the UCA cream and SI cream groups were significantly lower than those of the C (+), HO, PG, and Ac groups.

Six hours after UV irradiation, the erythema score for the UNFs group was significantly lower than those of the C (+), HO cream, PG cream, Ac cream, ANFs cream, and UCA cream groups. The erythema score for the SI group was significantly lower than those of the C (+), HO, PG cream, Ac cream, and ANFs cream groups.

Twelve hours after UV irradiation, the erythema scores for both the UNFs cream and SI cream groups were significantly lower than those of the C (+), HO, PG cream, Ac cream, and ANFs cream groups.

Twenty-four hours after UV irradiation, the erythema score for the UNFs cream group was significantly lower than the scores for the C (+), HO cream, PG cream, Ac cream, and ANFs cream groups. The erythema score for the SI cream group was significantly lower than those of the C (+) and HO groups.

### 2.2. Sunburn Cell Counts in the Epidermis

Within hours after UVB irradiation, the skin shows reddening, swelling, and vesicle formation, which is referred to as a “sunburn”. Apoptosis occurs as a result of DNA fragmentation due to UV irradiation [26,27,28,29], and such apoptotic cells are called “sunburn cells”. Sunburn cells are characterized by cytoplasm with concentrated nuclei and an increase in eosinophilic staining [30]. 

The presence of sunburn cells in the epidermis was evaluated using specimens prepared by TdT-mediated dUTP nick end labeling (TUNEL) staining. TUNEL staining is a technique that facilitates visualization of fragmented DNA by using terminal deoxynucleotidyl transferase and biotinylated deoxyuridine triphosphate [31]. Thus, using TUNEL staining, we identified cells that became apoptotic as a result of UVB irradiation. The number of TUNEL-positive sunburn cells present between granular cells was counted.

**Figure 3:**
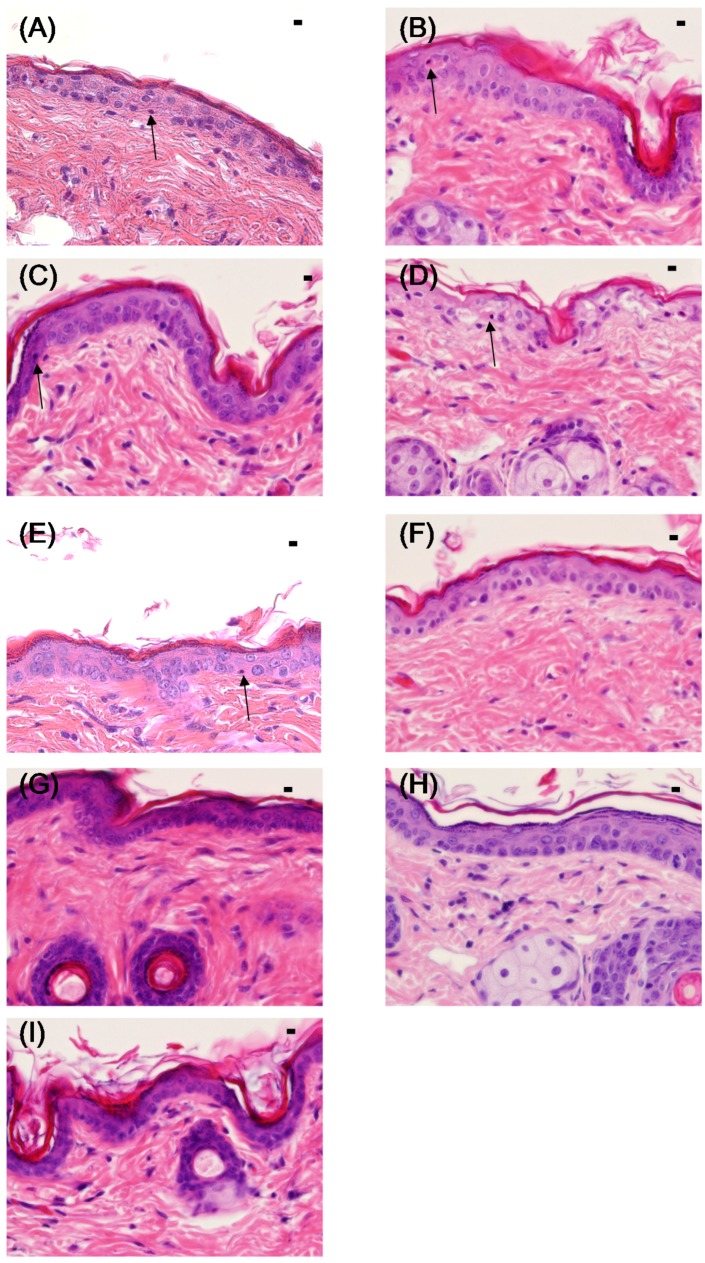
Histology (hematoxylin-eosin (H&E) staining) of the skin of hairless mice 24 h after UV irradiation (302 nm, 150 mJ/cm^2^). (**A**) C (+) (non-coated, irradiated) group; B: HO (hydrophilic ointment)-coated group; (**C**) PG (polyethylene glycol) cream-coated group; (**D**) Ac (aqueous acetic acid) cream-coated group; (**E**) ANFs (acetic acid chitin nanofibrils) cream-coated group; (**F**) UCA (aqueous urocanic acid) cream-coated group; (**G**) UNFs (urocanic acid chitin nanofibrils) cream-coated group; (**H**) SI (squid ink) cream-coated group; and (**I**) C (−) (non-coated and non-irradiated) group. Each specimen was subjected to H&E staining and photographed at a magnification of 400×. Scale bar = 100 μm. The sunburn cell in the epidermis is indicated above the figures with an arrow.

**Figure 4:**
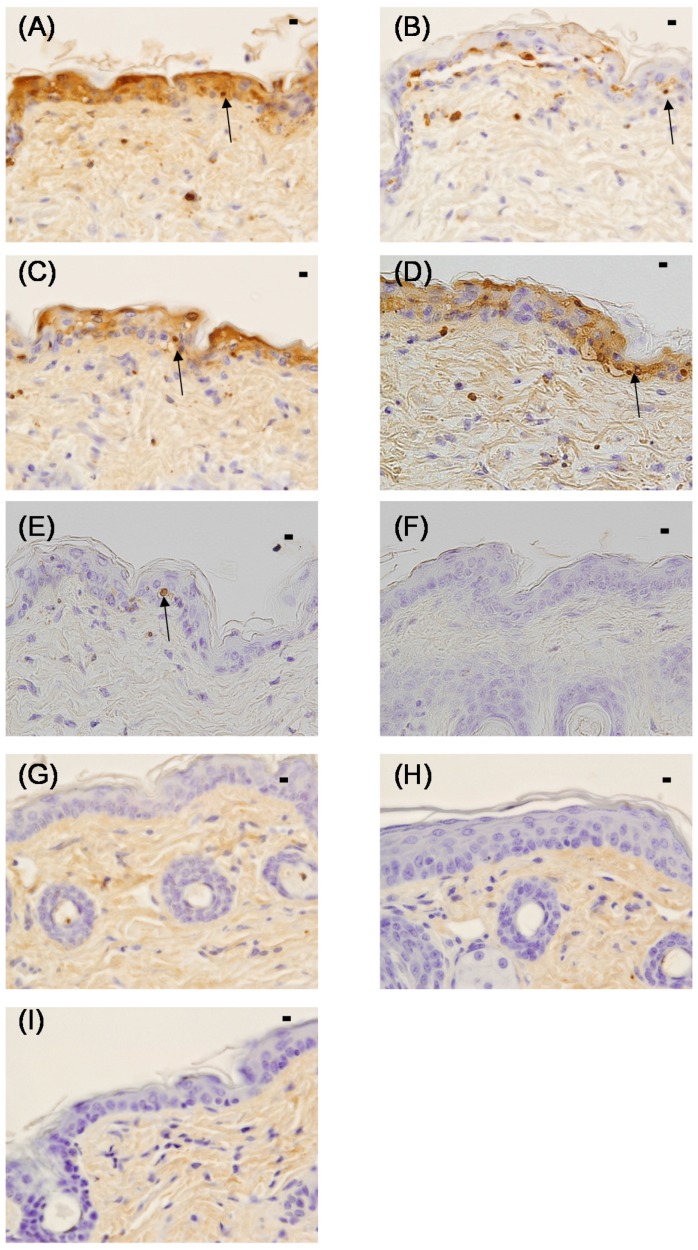
Histology (TUNEL staining) of the skin of hairless mice 24 h after UV irradiation (302 nm, 150 mJ/cm^2^). (**A**) C (+) (non-coated, irradiated) group; (**B**) HO (hydrophilic ointment)-coated group; (**C**) PG (polyethylene glycol) cream-coated group; (**D**) Ac (aqueous acetic acid) cream-coated group; (**E**) ANFs (acetic acid chitin nanofibrils) cream-coated group; (**F**) UCA (aqueous urocanic acid) cream-coated group; (**G**) UNFs (urocanic acid chitin nanofibrils) cream-coated group; (**H**) SI (squid ink) cream-coated group; and (**I**) C (−) (non-irradiated non-coated) group. Each specimen was subjected to TUNEL staining and was photographed at a magnification of 400×. Scale bar = 100 μm. The sunburn cell in the epidermis is indicated above the figures with an arrow.

Figure 3 shows a picture of H&E-stained cutaneous tissue at 24 h after UV irradiation, and Figure 4 shows the TUNEL staining of the same tissues. In the C (+) group, the boundary between the dermal layer and the basal layer of the epidermis was unclear, and sunburn cells with eosinophilic cytoplasm and aggregated nuclei were observed (Figure 3). The histological findings for the SI cream group were similar to those for the C (−) group: the boundary between the dermal and basal layers of the epidermis was clear, and no sunburn cells were detected. The histological findings for the HO, PG cream, and Ac cream groups were similar to those for the C (+) group, and the presence of sunburn cells was confirmed.

The presence of sunburn cells could not be confirmed in the UCA cream and UNFs cream groups, and the boundary between the dermal and basal layers of the epidermis was clear. In the ANFs cream group, the boundary between the dermal and basal layers of the epidermis was slightly unclear; however, very few sunburn cells could be detected.

In the C (+), HO, PG cream, Ac cream, and ANFs cream groups, TUNEL-positive sunburn cells were observed from the basal layer of the epidermis to the stratum spinosum (Figure 4). In the C (+) group, sunburn cells were observed in large numbers; however, they were not found in the SI group. Similar numbers of sunburn cells were observed in the HO, PG cream, Ac cream, and ANFs cream groups. Sunburn cells were present in the ANFs cream group; however, they were nearly absent in the UCA cream and UNFs cream groups.

**Figure 5:**
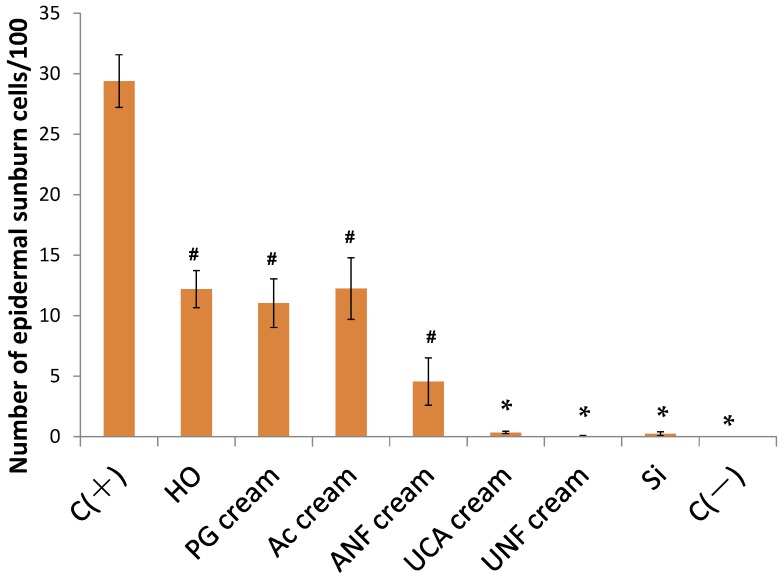
Quantification of sunburn cells in the skin of hairless mice 24 h after UV irradiation (302 nm, 150 mJ/cm^2^). The sunburn cell counts in the UC cream, UNFs cream, SI cream, and C (−) groups were significantly different from those in the C (+), HO, PG cream, and Ac cream groups. The sunburn cell counts in the HO, PG cream, Ac cream, and ANFs cream groups were significantly different from those in the C (+) group. The data shown are the mean ± SE. * *p* < 0.01, significantly different from C (+), HO, PG, and Ac. **^#^** *p* < 0.01, significantly different from C (+), (Scheffé’s *F*-test).

The sunburn cell counts determined from the TUNEL staining are shown in Figure 5. The sunburn cell counts in the UCA, UNFs, SI, and C (−) groups were significantly lower than those of the C (+), HO, PG cream, and Ac cream groups. The sunburn cell counts in the HO, PG cream, Ac cream, and ANFs cream groups were significantly lower than those of the C (+) group.

### 2.3. Conclusions in These Two Results

In this study, we examined the protective effects of various formulations against UV radiation, with a focus on UV irradiation-induced erythema and sunburn cells. The sunburn cell count results demonstrated that the UNFs cream, UCA cream, and SI cream significantly and equivalently prevented the generation of sunburn cells. The erythema scores showed differences among the UNFs cream, UCA cream, and SI cream groups; however, these differences were not significant. Compared with UCA cream and SI cream, which was used as a positive control, UNFs cream controlled erythema. UNFs cream inhibited erythema more conspicuously than UCA cream. The UCA cream erythema score rose over time, but UNFs cream maintained approximately the same low score from 2 h to 12 h. Thus, the UNFs cream inhibited erythema by UV irradiation better than the UCA cream did. Moreover, although the differences were not significant, we obtained interesting results with the ANFs cream. ANFs cream controlled the generation of sunburn cells and erythema at 2, 4, and 6 h when compared with the C (+) group, as well as with the HO, PG cream, and Ac cream groups. The results of our previous study suggested that NFs has anti-inflammatory effects. When we measured the levels of secreted cytokines from the skin of HR-1 mice treated with NFs using Franz cells, we noted that there was less IL-1α and TGF-β secreted from NFs-treated samples than from the control [32]. Inflammatory cytokines such as IL-1α induce the expression of COX-2 and increase the number of PGs. It has been suggested that NFs controls the expression of COX-2 by controlling inflammatory cytokines, which suppresses inflammation of the skin and reduces erythema. In addition, various studies have shown the antioxidant effects of chitin. Ngo *et al.* [33] determined that chitin oligomers released by the acidic hydrolysis of crab chitin had inhibitory effects on myeloperoxidase (MPO) activity in human myeloid cells, and their direct radical scavenging effect and intracellular glutathione levels increased significantly in a time-dependent manner [33]. Azuma *et al.* [34] evaluated the anti-inflammatory effects of orally administered NFs in a mouse model of experimental inflammatory bowel disease (IBD). The number of MPO-positive cells in the submucosal layer was significantly lower in the NFs group than in the control group [34]. These results suggest that chitin and NFs act as antioxidants. The results of our study suggest that the antioxidant effect of NFs may reduce DNA damage and ROS production due to UV irradiation and controls the generation of sunburn cells. They also suggest that UNFs is a superior sun protection material to UCA alone.

## 3. Experimental Section

### 3.1. Test Specimens

As shown in Table 1, mice were divided into seven experimental groups and treated with the following substances: (1) Acetic acid (Ac) cream (1 wt % Ac aqueous solution mixed with hydrophilic ointment (HO; NIPRO Pharma Corporation Co., Ltd., Osaka, Japan) at a ratio of 1:1); (2) HO (HO only); (3) Polyethylene glycol (PG) cream (35 wt % PG aqueous solution (NOF Corporation, Tokyo, Japan) mixed with HO at a ratio of 1:1); (4) Acetic acid chitin nanofibrils (ANFs) cream (0.82 wt % ANFs and 99.18 wt % (1 wt % Ac) aqueous solution mixed with HO at a ratio of 1:1); (5) UCA cream (0.2 wt % UCA and 35 wt % PG as an aqueous solution mixed with HO at a ratio of 1:1); (6) UNFs cream (1 wt % UNFs and 99 wt % (0.2 wt % UCA and 30 wt % PG) as an aqueous solution mixed with HO at a ratio of 1:1); and (7) Squid ink (SI) cream (1 wt % SI and 35 wt % PG in an aqueous solution mixed with HO at a ratio of 1:1) (Table 1).

**Table 1:** Composition of test specimens (%).

Substances	1 wt % Ac	HO	35 wt % PG	ANFs	0.2 wt% UCA in 35 wt% PG	0.2 wt % UCA In 30 wt % PG	UNFs	1 wt% SI in 35 wt % PG
Ac cream	50	50						
HO		100						
PG cream		50	50					
ANFs cream	49.59	50		0.41				
UCA cream		50			50			
UNFs cream		50				49.5	0.5	
SI cream		50						50

Acetic acid (Ac) cream: 1 wt % Ac aqueous solution mixed with hydrophilic ointment (HO) at a ratio of 1:1; HO: HO only; Polyethylene glycol (PG) cream: 35 wt% PG aqueous solution mixed with HO at a ratio of 1:1; Acetic acid chitin nanofibrils (ANFs) cream: 0.82 wt % ANFs and 99.18 wt % (1 wt % Ac) as an aqueous solution mixed with HO at a ratio of 1:1; UCA cream: 0.2 wt % UCA and 35 wt % PG as an aqueous solution mixed with HO at a ratio of 1:1; UNFs cream: 1 wt % UNFs and 99 wt % (0.2 wt % UCA and 30 wt % PG) as an aqueous solution mixed with HO at a ratio of 1:1; Squid ink (SI) cream: 1 wt % SI and 35 wt % PG in an aqueous solution mixed with HO at a ratio of 1:1.

The samples were prepared according to the following methods: Ac cream was prepared in distilled water at a final concentration of 1 wt %. Next, 1 mL of the aqueous solution was mixed with 1 g of HO. To prepare the PG cream, an aqueous solution of PG was prepared by mixing 84 g of an aqueous solution of 40 wt % PG (molecular weight 20,000) with 12 g of distilled water. Then 1 mL of the aqueous solution was mixed with 1 g of HO. To prepare the ANFs cream, 197 g of purified water was added to 2 g of crab shell-derived chitin powder (Nacalai Tesque, Inc., Kyoto, Japan, deacetylation degree: <5%) and stirred overnight. The aqueous solution was subjected to 30 passes on a Star Burst system (Star Burst Mini, HJP-25001S; Sugino Machine Co., Ltd. Toyama, Japan) at 245 MPa. Finally, 1 mL of the aqueous solution was mixed with 1 g of HO. To prepare the UCA cream, a PG aqueous solution was prepared by mixing 84 g of an aqueous solution of 40 wt % PG with 12 g of distilled water. Next, 0.192 g of urocanic acid (Sigma-Aldrich, St. Louis, MO, USA) was added to the aqueous solution and stirred until it dissolved. Finally, 1 mL of the aqueous solution was mixed with 1 g of HO. To prepare the UNFs cream, 2 g of urocanic acid was added to 1 L of distilled water and dissolved by heating at 40 °C. Ten grams of crab shell-derived chitin powder was added to the urocanic acid aqueous solution, and the mixture was used as a chitin suspension. The suspension was defibrated twice using a millstone grinder (MKCA6-3; Masuko Sangyo Co., Ltd., Saitama, Japan) at 1500 rpm. An aqueous solution of 30 wt % PG was added to this sample. Then 1 mL of the aqueous solution was mixed with 1 g of HO. To prepare the SI cream, 60 g of distilled water was added to 40 g of PG, and the mixture was dissolved to obtain an aqueous solution of 40 wt % PG. Next, 12 g of ikasumi melamine (melanin density 8 wt %) was added to 84 g of an aqueous solution of 40 wt % PG and stirred. The aqueous solution was adjusted so that it would contain 1 wt % ikasumi melamine and 35 wt % PG. Finally, 1 mL of the aqueous solution was mixed with 1 g of HO. The UCA contained *trans*-urocanic acid.

### 3.2. Experimental Animal Model

Male 8–9-week-old Hos:HR-1 mice (25–35 g, Hoshino Laboratory Animals Inc., Ibaraki, Japan) were used. All animal care and experimentation procedures were approved by the Animal Research Committee of Tottori University.

### 3.3. Experimental Groups

The mice were divided into nine groups: The Ac cream group (*n* = 5), HO (*n* = 5), PG cream (*n* = 5), ANFs cream (*n* = 5), UCA cream (*n* = 5), UNFs cream (*n* = 5), SI cream (*n* = 5), C (+) (non-coated, irradiated; *n* = 5), and C (−)(non-coated, non-irradiated; *n* = 5) groups. 

### 3.4. Sample Application and UV Irradiation

Each sample (2 g) was applied to the left side of the back of the test mice. In addition to the seven sample groups, two control groups were included in this study. In one group, no sample was applied to the mice, but they were irradiated (the positive control, C (+)), and in another group, no sample was applied and the mice were not irradiated (C (−)). All of the groups were irradiated (150 mJ/cm^2^) with a UV crosslinker (Model CL-1000M, 302 nm UV; UVP, USA). In accordance with the criteria established by the OECD, erythema of the skin was assessed at 2, 4, 6, 12, and 24 h after irradiation.

### 3.5. Preparation of Irradiated Samples

Twenty-four hours after UV irradiation, the animals were euthanized by cervical dislocation, and the skin at the irradiated site was sampled using a Dermapunch (8 mm; Nipro Co., Osaka, Japan). The collected skin samples were immersed and fixed in 10% formalin (Mildform 10N; Wako Pure Chemical Industries Ltd., Osaka, Japan). The skin surface samples were cut into 4-μm thick vertical slices for cross-sectional observation. Histological observations were performed after hematoxylin-eosin (H&E) and TUNEL staining.

### 3.6. Evaluation Methods

The erythema score was determined as the mean score of two observers, in accordance with OECD standards, at 2, 4, 6, 12, and 24 h after UV irradiation at the site of sample application. The presence of sunburn cells in the epidermis was evaluated using specimens prepared by TUNEL staining. Basal cells (*n* = 100) inside the epidermis were counted (excluding hair follicle cells), and the number of TUNEL-positive sunburn cells present between granular cells was counted. These measurements were conducted at four random times, and the mean number of sunburn cells per 100 cells was recorded.

### 3.7. Statistical Analysis

Data analysis was performed using 4-step Excel Statistics (OMS Publishing, Saitama, Japan). For each experiment, we performed Bartlett’s test for normality. For those where we confirmed normality or equal variance, single-factor ANOVA was performed; otherwise, we used the Kruskal-Wallis test. Afterwards, a multiple comparison test (Tukey-Kramer test or Scheffé’s F-test) was performed. *P* values less than 0.05 were considered statistically significant, and *P* values less than 0.01 were considered highly significant.

## 4. Conclusions

This study demonstrated that UNFs has a protective effect against UVB irradiation and inhibits UVB irradiation-induced erythema and sunburn cell generation. In addition, the results suggested that NFs itself has a protective effect against UVB, and that the anti-inflammatory and antioxidant effects of NFs affect its protective effect against UVB. This study also showed that UNFs has a protective effect against UVB, and inhibits UVB irradiation-induced erythema and sunburn cell generation. Likewise, the results suggested that NFs has a protective effect against UVB, and that the anti-inflammatory and antioxidant effects of NFs may affect its protective effect against UVB. A combination of NFs and UCA might show a greater Anti-UV effect than conventional preparations do. Further studies are needed to elucidate the underlying mechanism, investigate the anti-inflammatory and antioxidant effects of UNFs, and digitize the UV-ray absorbency of UNFs.
